# Comparison of bacterial filtration efficiency vs. particle filtration efficiency to assess the performance of non-medical face masks

**DOI:** 10.1038/s41598-022-05245-4

**Published:** 2022-01-24

**Authors:** Henrietta Essie Whyte, Yoann Montigaud, Estelle Audoux, Paul Verhoeven, Amélie Prier, Lara Leclerc, Gwendoline Sarry, Coralie Laurent, Laurence Le Coq, Aurélie Joubert, Jérémie Pourchez

**Affiliations:** 1 Mines Saint-Etienne, Université Lyon, Université Jean Monnet, INSERM, U 1059 Sainbiose, Centre CIS, 42023 Saint-Etienne, France; 2 IMT Atlantique, CNRS, GEPEA, UMR 6144, 4 rue Alfred Kastler, 44307 Nantes, France; 3 CIRI (Centre International de Recherche en Infectiologie), GIMAP Team, University of Lyon, University of St-Etienne, INSERM, U1111, CNRS UMR5308, ENS de Lyon, UCB Lyon 1, St-Etienne, France; 4 Laboratory of Infectious Agents and Hygiene, University Hospital of St-Etienne, St-Etienne, France

**Keywords:** Biomedical engineering, Biological physics, Chemical physics, Bacteria

## Abstract

As a result of the current COVID-19 pandemic, the use of facemasks has become commonplace. The performance of medical facemasks is assessed using Bacterial Filtration Efficiency (BFE) tests. However, as BFE tests, require specific expertise and equipment and are time-consuming, the performance of non-medical facemasks is assessed with non-biological Particle Filtration Efficiency (PFE) tests which are comparatively easier to implement. It is necessary to better understand the possible correlations between BFE and PFE to be able to compare the performances of the different types of masks (medical vs. non-medical). In this study BFE results obtained in accordance with the standard EN 14683 are compared to the results of PFE from a reference test protocol defined by AFNOR SPEC S76-001 with the aim to determine if BFE could be predicted from PFE. Our results showed a correlation between PFE and BFE. It was also observed that PFE values were higher than BFE and this was attributed to the difference in particle size distribution considered for efficiency calculation. In order to properly compare these test protocols for a better deduction, it would be interesting to compare the filtration efficiency for a similar granulometric range.

## Introduction

The world is currently in the midst of the COVID-19 pandemic, which is caused by the severe acute respiratory syndrome coronavirus-2 (SARS-CoV-2) virus. The transmission of this virus is widely thought to be by aerosols and droplets. Droplet transmission and airborne transmission constitute the major routes of person-to-person transmission^1,2^. Despite the continued scientific debate on the dominant mode of transmission, recent studies have shown that airborne transmission is likely the dominant route of transmission especially in the indoor environment with low levels of ventilation^3–5^. In a bid to reduce the spread of the virus, countries across the globe have issued regulations such as lockdowns (with the aim of restricting movement and mass social interactions) along with recommendations on social distancing, hand hygiene, and the use of face covering/masks^6^.

Since early 2020, the use and demand of facemasks has considerably increased during this COVID pandemic^7^. The use of masks has been shown to help reduce the spread of the virus and protect wearers from contracting COVID-19^8^. In a study of non-health workers, Liang et al.^9^ found that wearing masks provided a significant protection effect and reduced transmission cases by 47%. Worby and Chang^10^ used mathematical modeling to examine the epidemiological impact of facemasks and found that facemasks can reduce SARS-CoV-2 transmission. Finally, in a retrospective analysis of confirmed COVID-19 cases in Beijing, the risk of transmission was lower in households where one or more family members wore a mask, compared to those where no one did^11^.

The term “facemask” generally refers to protective equipment with the primary function of reducing the transmission of airborne particles or droplets. Facemasks are used with the intention of preventing the infected wearer transmitting the virus to others (source control) and/or to offer protection to the healthy wearer against infection (protection). There are three main categories of face masks; (1) surgical masks, (2) face filtering pieces (e.g. FFPs or N95) and non-medical or community face coverings. surgical and non-medical masks intended to prevent droplets emitted by the wearer from being projected onto the surrounding area. Finally, FFP masks are used to protect the wearer against the inhalation of both droplets and airborne particles. Surgical masks are regulated as medical devices and FFP masks as respiratory protection device and are designed to meet more demanding performance criteria. In their work to evaluate the survival rate of bio aerosols on filter layers of FFPs and surgical masks, Jeong et al.^12^ showed that FFPs were more effective in physically blocking bio aerosols and thus showed lower portion of microbial viability compared to surgical masks.

By contrast, non-medical or community face coverings are commercial, home-made, and improvised masks that vary considerably in design, material, and construction and then in filtration performance. However, their public use has become widely accepted by several countries in a bid to address worldwide shortage of medical masks brought on by the pandemic^13,14^.

In Europe, medical masks must conform to the standard EN 14683:2019^15^. Based on the Bacterial Filtration Efficiency (BFE) parameter, the medical masks are classified into two types. Type I masks have a BFE ≥ 95% and Type II masks have a BFE ≥ 98%. The FFP masks are regulated by the standard EN149^16^. The standard specifies several performance criteria among which are the particle filtration efficiency (PFE), breathability, leakage and CO_2_ content of inhalation air. According to the PFE, FFPs can be classified into three types. FFP1 have PFE ≥ 80%; FFP2 have PFE ≥ 94% and FFP3 have PFE ≥ 99%. With regards to the non-medical or community face coverings, the European committee for standardization (CEN) developed a guideline that presents manufacturing specifications and testing protocols (breathability and filtration efficiency) ; CWA 17553:2020^17^. The objective was to allow the general public to manufacture and distribute these masks certified by public and private laboratories that are capable of conducting the testing protocols. The CWA 17553:2020 establishes two categories based on the filtration efficiency (FE) of particles to around 3 ± 0.5 µm; category 1 have FE ≥ 90% and category 2 have FE ≥ 70%. The regulation however doesn’t impose a filtration efficiency test protocol and allows producers to use either existing European standards listed in the document (EN14683, EN 13274-7, etc.) or available methodologies developed by CEN members listed in the document such as AFNOR SPEC S76-001 for France. In link with the specifications, only the filter material has to be characterized and not the entire mask. Leakage tests are reserved only for face filtering pieces (FFP and N95).

The high demand for these masks (medical and non-medical) brought on by the current pandemic situation, consequently leads to a high demand for conformity testing of masks. Currently, few laboratories are equipped with the necessary equipment to conduct bioaerosol tests to assess the BFE parameter on these masks. Additionally, the standard EN 14683:2019 test protocol requires specific expertise and equipment and is time consuming. It has been established that non-biological particles and biological particles are captured by similar mechanisms^18,19^. In this regard, PFE tests are a relatively easier alternative to implement. This cheaper and faster filtration test protocols using non-biological aerosol (by contrast with test protocols using bioaerosol, i.e. droplets containing bacterial species) makes mask testing more accessible and thus increase the number of laboratories that can evaluate mask filtration performance. Nevertheless, these non-biological aerosol test protocols must still guarantee the security and performance of these masks. In this context, the aim of this article is to compare a posteriori without any additional experiments, the performances of non-medical masks in order to determine whether the BFE can be deduced from the PFE results. Thus, we studied the possible correlation between results of non-biological PFE from a reference test protocol defined by AFNOR SPEC S76-001 to those from BFE tests (according to EN14683) for different non-medical masks. In this work, the focus is on filtration efficiency of the masks for a mean particle size of 3.0 µm with regards to exhaled droplet. There are however, numerous studies aiming to establish the droplet size of greatest concern regarding the SARS COV-2 aerosols^20–25^. The choice to compare results of BFE and PFE at 3 µm is made purely to be in accordance with the AFNOR SPEC S76-001 and the EN14683.

## Materials and methods

The materials and test protocols used in the work are presented in the following sections.

### Masks

The filtering materials of four non-medical masks were tested in this study. The masks were made from different fabrics produced by French manufacturers. Throughout this article, the masks will be referred to as A, B, C and D. The breathing performance was measured as the air permeability for a vacuum pressure of 100 Pa. The results for the four mask samples are Table 1. It is observed that the air permeability covers a large range from 100 to 300 L m^−2^ s^−1^.

**Table 1:** Permeability of 4 masks models used in this study.

Mask	Permeability at 100 Pa (L m^−2^ s^−1^)
A	203.0
B	99.3
C	154.0
D	309.0

### Particle filtration efficiency (PFE) test protocol

**Figure 1:**
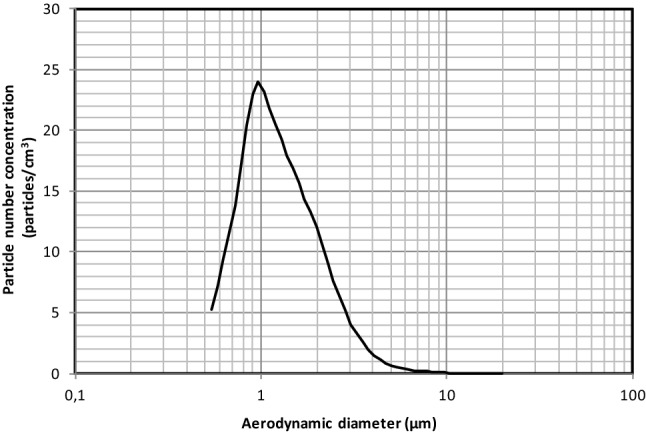
Size distribution of Holi particles.

The PFE protocol used in this study was developed by the French Defense Procurement Agency (DGA). The DGA is one of the agencies that is able to test the performance of non-medical masks. The PFE values evaluated in this study were obtained from the DGA reports provided to the manufacturers. The experimental setup consists of a test bench which allowed the generation and measurement of aerosols. Poly-disperse Holi particles were generated with a RBG 1000 PALAS. The Holi particles were generated with a median diameter of 1.1 µm (GSD of 1.6) and an average total concentration of 350 particles cm^−3^ (Fig. 1). A mask sample (cut out from full mask) was placed in a sample holder which were fabricated in-house and had an internal diameter of 44 mm.

Two aerodynamic particle sizers (APS, model 3321; TSI Inc.) and two sample holders (one with mask sample and the other one without) were used to measure simultaneously aerosol number concentrations upstream and downstream of the sample holder.

The particle size considered for filtration efficiency is fixed at 3 µm as per the AFNOR SPEC S76-001. The PFE is calculated as:

$$PFE=1- \frac{{C}_{down}}{{C}_{up}}* 100$$

where *C*_*down*_ and *C*_*up*_ are the particle concentrations downstream and upstream the mask sample respectively; a correction factor was applied on concentrations ratio to take into account the systematic bias between the two measurement channels. The correction factor was calculated with data from a test without the sample on the sample holders. The PFE value is the average of three measurements of the same sample.

### Bacterial filtration efficieny (BFE) test protocol

**Figure 2:**
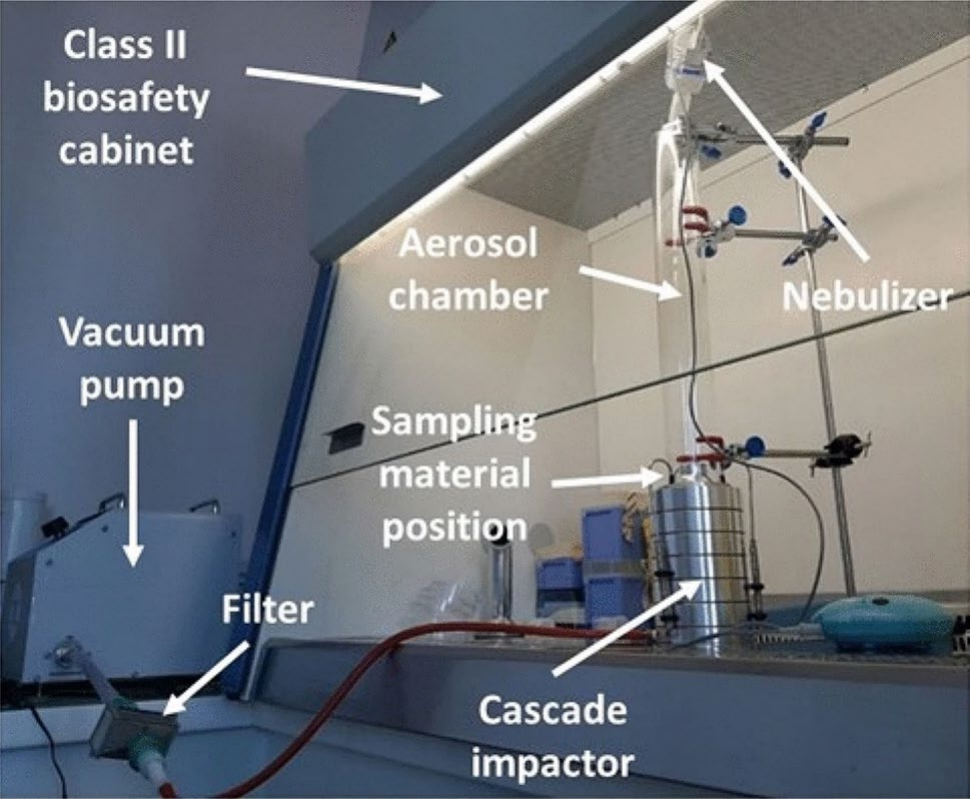
BFE experimental set-up developed by CIS of Mines Saint-Etienne.

The BFE tests were performed by the accredited laboratory of the Centre Ingénierie et Santé (CIS) of Mines Saint-Etienne. The test protocol is compliant with the EN14683:2019 standard test method. The experimental set-up previously described in^26^ and^27^ is shown in Fig. 2.

Briefly, the mask sample was clamped between an aerosol chamber (glass tube 60 mm in external diameter and 60 mm long) and a six-stage viable aerosol cascade impactor (ACI) (Tisch Environmental, Cleaves, USA) requiring a flowrate of 28.3 L min^−1^. The 50% effective cut-off diameters (i.e., the particle diameters corresponding to 50% sampling efficiency) for each of the six stages when operating at 28.3 L min^−1^ are 7 µm (stage 1), 4.7 µm, 3.3 µm, 2.1 µm, 1.1 µm and 0.65 µm (stage 6).

Each sample measured at least 100 mm × 100 mm and the test area was therefore at least 49 cm^2^ as required by EN 14683. The tests were performed by putting the interior of the mask in contact with the aerosolized bacteria. Each sample was conditioned at 21 ± 5 °C and 85 ± 5% relative humidity for at least 4 h to reach atmospheric equilibrium prior to testing.

A bacterial culture of *Staphylococcus aureus* (ATCC 29213) was inoculated into 30 ml tryptic soy broth in an Erlenmeyer flask and incubated with mild shaking at a temperature of (37 ± 2) °C for (24 ± 2) h. The counts are expressed in Colony Forming Units (CFU). The culture medium was then diluted to obtain a concentration of approximately 5 × 10^5^ CFU ml^−1^ for the tests. The inoculum was stored in a freezer at − 80 °C. The bacteriological inoculum was maintained on average between 1.7 × 10^3^ CFU and 3.0 × 10^3^ CFU as required in EN 14683. The EN 14683 also imposes a mean particle size (MPS) of 3.0 ± 0.3 µm. The bacterial challenge corresponds to the total CFU observed on the six positive-control dishes.

$$MPS= \frac{\left(P1* C1\right)+\left(P2* C2\right)+\left(P3 * C3\right)+\left(P4 * C4\right)+\left(P5* C5\right)+\left(P6 * C6\right)}{C1+C2+C3+C4+C5+C6}$$

Px is the the 50% effective cut-off diameters of each of the six stages, Cx is the viable particle counts obtained from each of the six agar collection surfaces and x is the stage number (1–6).

The bacteriological inoculum was nebulized using an E-Flow mesh nebulizer (Pari GmbH, Starnberg, Germany) for 1 min and then the air flow in the cascade impactor was maintained for an additional minute (total test duration was 2 min). To evaluate the BFE of a mask, a series of eight successive measurements must be performed. First, a positive-control run was performed without a mask positioned between the cascade impactor and aerosol chamber. Next, five experiments were performed on test samples, changing the mask for each experiment and cleaning the experimental set-up to avoid bacterial contamination. A second positive control experiment was then performed. Finally, this cycle of eight consecutive experiments ended with a negative-control run in which air is passed, without adding bacteria, through the cascade impactor for 2 min (this served as a contamination control to verify that the bacteria deposited during the positive run and the test samples came only from the bioaerosol source).

In an effort to provide insight on the possible modernization of the standards on BFE analysis, Pourchez et al.^26^*.* showed that: (i) 90-mm disposable Petri dishes can be used instead of the 100-mm dishes supplied with the six-stage viable ACI and (ii) Automatic HD colony counters can be used to directly count viable particles on collection substrates without requiring the use of the positive-hole conversion table. In this work, 90-mm disposable Petri dishes were used. The petri dishes were removed and incubated at 37 ± 2 °C for 22 ± 2 h. The CFU were counted with an automatic colony counter Scan 1200 (Interscience).

The BFE is calculated as:

$$BFE= \frac{C-T}{C * 100}$$

where *C* is the mean of the two positive runs of the total of the six plate counts, and *T* is the total of the six plate counts for each test sample. Five samples each are tested for Masks A and B, 4 samples for Mask C and 3 samples for Mask D.

### Statistical analysis

Statistical analyses were performed with GraphPad Prism 8.4.2 (GraphPad Software, San Diego, CA, USA). A conformity analysis of the BFE to the 95% minimum value for Type I masks (EN 14683) was performed using the student t-test. In order to evaluate if there were any significant differences between the filtration efficiency results of the PFE test method and the BFE test method a Wilcoxon test was used. p-values < 0.05 were considered significant. Correlation coefficients for the comparison of filtration efficiency between the test methods were obtained using Excel.

## Results and discussion

### Analysis of conformity of masks according to AFNOR SPEC S76-001 and EN 14683

According to the standard EN 14683, a mask is deemed compliant when the BFE is ≥ 95% (Type I) whilst for AFNOR SPEC S76-001, a mask is complaint when PFE ≥ 90% (Category 1). The PFE and BFE results of the masks were statistically compared to these limits in order to determine their compliance. Table 2 presents the percentage of particle and bacterial filtration efficiency for the four non-medical mask models.

**Table 2:** Conformity of masks according to AFNOR SPEC S76-001 Category 1 and EN14683 Type I limit.

MASK	PFE (%)	BFE (%)
A	98.80 Compliant—category 1 AFNOR SPEC S76-001	94.16 ± 1.79 at compliance limit – Type I EN14683
B	99.50 Compliant—category 1 AFNOR SPEC S76-001	95.73 ± 0.18 compliant—Type I EN14683
C	98.00 compliant—category 1 AFNOR SPEC S76-001	93.93 ± 0.41 non-compliant—Type I EN14683
D	94.00 Compliant—category 1 AFNOR SPEC S76-001	92.67 ± 1.45 non-compliant—Type I EN14683

The PFE values obtained ranged from 94.0 to 98.8% whilst BFE values ranged from 92.67 to 95.73%. Considering the PFE values, all the masks were classified as conforming to category 1 (PFE > 90%) according to AFNOR SPEC S76-001. Considering the BFE values, only the Mask B can be classified as conforming to type I (BFE > 95%) according to EN14683 as its BFE was significantly higher than the limit of 95%. The BFE values of Masks C and D were significantly lower than the BFE limit of 95% and were thus classified as non-compliant. The BFE of Mask A was not significantly different from the BFE limit of 95%, therefore was within the limit of conformity. Statistically this mask can be classified as compliant conforming to type I (BFE > 95%) according to EN14683. As indicated earlier, the BFE value presented for Mask A is the average value of 5 masks from the same lot (Table 3), for Mask A, 2 masks out of the 5 had efficiencies higher than 95% whilst 3 of the masks had efficiencies lower than 95%. This means that 60% of the masks from the same lot would not conform to type I (BFE > 95%) according to EN14683. In the case where these masks are supposed to ensure the safety of the wearer, regulatory agencies, as a precautionary measure, are more likely to reject the lot. In this regard, Mask A was deemed non-compliant.

**Table 3:** BFE of 5 sample mask of model Mask A.

Mask A	BFE (%)
1	95.39
2	96.65
3	93.43
4	92.44
5	92.88

### Possible correlation between PFE and BFE

**Figure 3:**
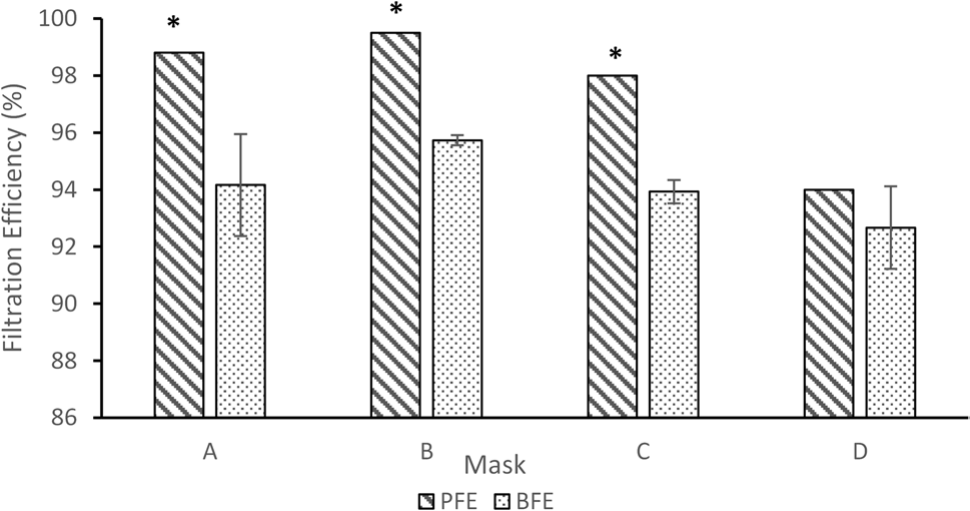
Comparison of filtration efficiencies between the PFE test protocol and BFE test protocol. *Significantly different from BFE.

Considering a comparison between the results from the two methods (Fig. 3), the BFE test results were always lower than the PFE test results irrespective of the model of mask with a difference ranging from 1.33 to 4.64%. The statistical comparison of the two tests showed that the median efficiency for 3 out of 4 masks using the PFE test protocol was significantly higher (p < 0.05) than efficiency values for the BFE test protocol. Masks A, B and C had significantly higher PFE values compared to BFE values whilst for Mask D, its PFE value wasn’t statistically different from the BFE value.

**Figure 4:**
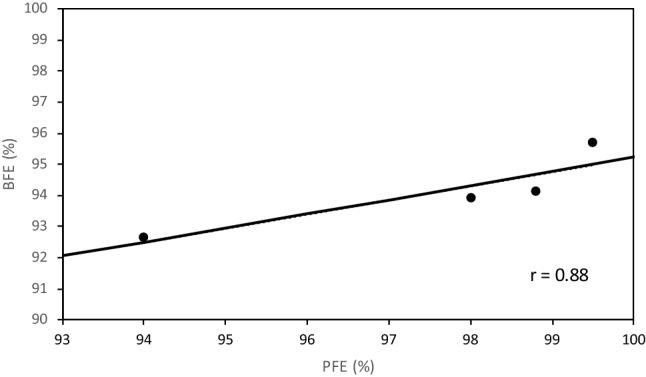
Correlation between PFE values obtained using the AFNOR SPEC S76-001 test protocol and BFE values obtained using the EN 14683 test protocol.

The BFEs obtained with the EN 14683 standard test were correlated with the PFEs obtained with the reference AFNOR SPEC S76-001 test. Even though the filtration efficiency is the most important safety parameter to determine the performance of the mask, the breathing performance of the masks measured by the air permeability is also important. The filtration efficiency should therefore be measured only if the masks are of acceptable breathing performance. In the AFNOR SPEC S76-001 protocol, the breathing performance must be higher than 96 L m^−2^ s^−1^ at 100 Pa and as seen in Table 1, all four masks ae compliant to this limit. The results (Fig. 4) showed that there was good correlation (r = 0.88) between BFE and PFE. It was also observed that whatever the filtration test (PFE or BFE), the same ranking of the masks in term of filtration performance was obtained: Mask B ≥ Mask A ≥ Mask C > Mask D which confirms correlation between the two test protocols.

### Identification of the main features which could contribute to the differences in terms of filtration results between the PFE and BFE methods

As previously shown, the PFE’s were always higher than the BFE’s for all four masks. This difference could be due to the differences in test conditions. The filtration efficiency of fibrous filter materials is influenced by factors such as the particle distribution efficiency, the aerosol charge and the face velocity^18,28–31^.

The PFE test protocol uses dry solid Holi particles whilst liquid *S. aureus* particles are utilized for the BFE test protocol. The behavior of the particles under wet or dry regime could potentially influence their ability to be captured and consequently influence the filtration efficiency. Filtration velocity is calculated at 5.5 cm s^−1^ for PFE test protocol and 9.6 cm s^−1^ for the BFE test protocol. Higher face velocities have been shown to reduce filtration efficiency^32–34^. However, at 3 µm, the filtration mechanism which is likely to be dominant is impaction which is favored by higher face velocities, consequently this parameter does not explain our results. The particle charge is another factor that influences the filtration efficiency. Neutralized aerosols are known to produce higher penetration (low efficiency) compared to charged aerosols^31^. The solid particles used in the PFE tests are slightly positively charged whilst the liquid particles used in the BFE test are slightly negatively charged. Joubert et al.^35^ and Han et al.^31^ showed that the polarity of a charged aerosol had no significant effect on the filtration efficiency.

Although these factors influence filtration efficiency, they do not explain the difference observed in this work. The main factor that potentially contributes to PFE > BFE for all four masks is the particle size distribution.

**Figure 5:**
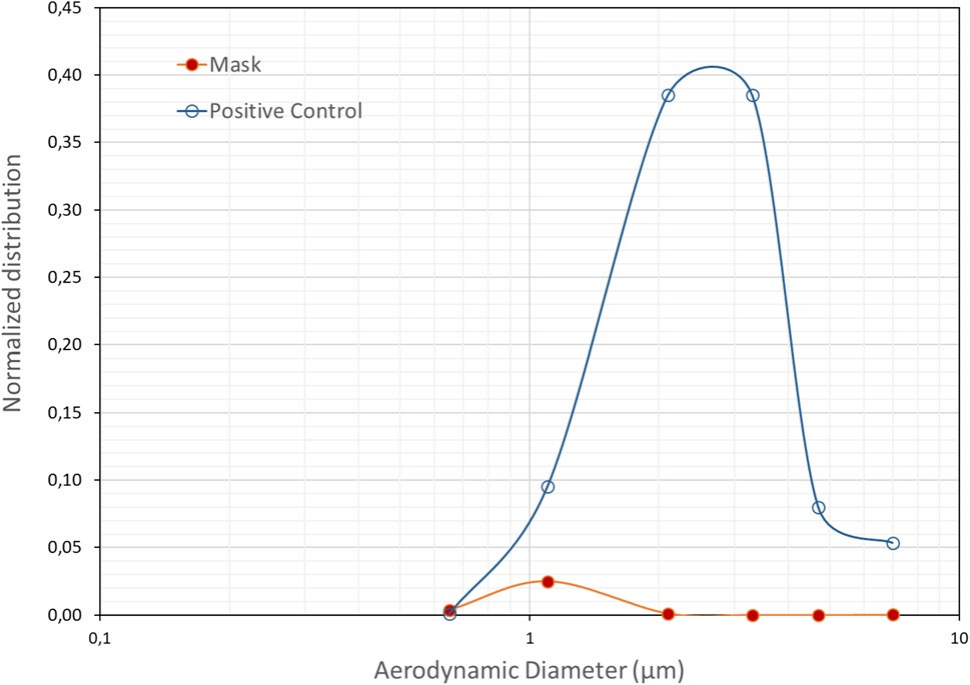
Normalized distribution of the *S. aureus* aerosol for positive control and the Mask A tests calculated as the ratio between the number of particles per stage and the total number of particles for the positive control test.

The particle size distribution is an important factor influencing filtration efficiency determination. The filtration efficiency is different for various size aerosols. The higher PFE values compared to BFE values could also be explained by the difference in the granulometric range of particle size used in the determination of the filtration efficiency. For both test methods studied in this work, the filtration efficiency is measured for polydispersed particles with average particle size around 3 µm. However, the granulometric range is different for both tests. The aerosol generated for the PFE test protocol is polydisperse but only 3 stages of the aerodynamic particle size (APS) channels with particle size of 3 ± 0.5 µm are exploited. The particle size range considered is therefore 2.74–3.4 µm. Concerning the BFE test protocol, as described in Sect. 2.3, the 3 µm imposed MPS is calculated for all six stages which gives a granulometric range of 0.65–7 µm with Geometric Standard Deviation (GSD) range typically between 1.38 and 2.2 as previously described^19^. All particles within this range are included in the determination of the BFE value contrary to the PFE test method where only particle sizes around 3 µm are considered. However, the filtration efficiency is not constant over the entire range. Besides, for all four masks, the normalized distribution (calculated as the ratio between the number of particles per stage and the total number of particles for the positive control test) of *S. aureus* aerosol particles shows that particle aerodynamic size below 1 µm (representing about 6–15% of the generated bioaerosol) accounts for 75- 90% of the particles that are able to penetrate the masks (Fig. 5).

## Conclusion

In this article, the Particle Filtration Efficiency (PFE) and the Bacterial Filtration Efficiency (BFE) for four non-medical face coverings were compared. The results showed some correlation between the two test methods with an identical classification of masks from the most performant to the least performant. Thus, it seems possible to assess from the PFE results, a plausible BFE value for a non-medical facemask, a posteriori without any additional experiments.

It was also observed that the values of the PFE were significantly higher than BFE for 3 of the masks. The difference was attributed the difference in test protocols mainly with regards to the particle size distribution (polydispersed versus diameter size around 3 µm for BFE and PFE respectively).

Considering the particle size distribution, although both tests provide a filtration efficiency at an average particle size of 3 µm, the range of interest in the determination of the filtration efficiency is very different and thus makes it less comparable. Whilst the PFE test method uses only the 3 channels of the aerodynamic particle sizer (APS) around 3 µm (2.74–3.40 µm), the BFE test method considers all stages of the six-stage viable Aerosol Cascade Impactor (0.65–7 µm). In order to properly compare these test protocols for a better deduction, it would be interesting to compare the filtration efficiency for a similar granulometric range.
